# Ethanol Reactions over FeMoO Using Low O_2_/Ethanol Molar Ratio: Reaction Network and Kinetics

**DOI:** 10.1021/acsomega.5c11700

**Published:** 2026-01-20

**Authors:** João G. R. Poço, Gustavo V. Olivieri, Elisabete M. Assaf, Reinaldo Giudici, Cláudio A. O. Nascimento

**Affiliations:** † Instituto de Pesquisas Tecnológicas do Estado de São Paulo, Departamento de Nanobiomanufatura, São Paulo, SP 05508-901, Brazil; ‡ Centro Universitário FEI, Departamento de Engenharia Química, São Bernardo Campo, SP 09850-901, Brazil; § Universidade de São Paulo, Instituto de Química de São Carlos, São Carlos, SP 13566-590, Brazil; ∥ Universidade de São Paulo, Escola Politécnica, São Paulo, SP 05508-010, Brazil

## Abstract

The search for sustainable paths for the valorization of ethanol to produce other substances, such as acetaldehyde, is an object of study in heterogeneous catalysis. This research investigates the conversion of ethanol using an iron–molybdenum oxide catalyst with low O_2_/EtOH molar ratios (0.0 and 0.05) in a Berty internal recycling reactor to clarify the dominant pathways and associated kinetics at an ethanol partial pressure above the flammability limit and to reveal the significant presence of dehydrogenation, dehydration reactions, and hydrogenation of ethylene to ethane. The catalyst characterization revealed the formation of the β-FeMoO4 phase, accompanied by an increase in specific area, pore volume, and pellet dimensions. The reaction network was also explored, and a kinetic model was developed and fit to experimental data, in order to estimate the kinetic parameters (kinetic constants and energies of activation) for the proposed reactions. Considering the low selectivity achieved for acetaldehyde with this catalyst under the studied conditions, it was concluded that it is not possible to apply an oxygen-distributed fed reactor. However, the experimental part, combined with the kinetic modeling, can contribute to further investigations on experimental conditions to enhance acetaldehyde production.

## Graphical abstract

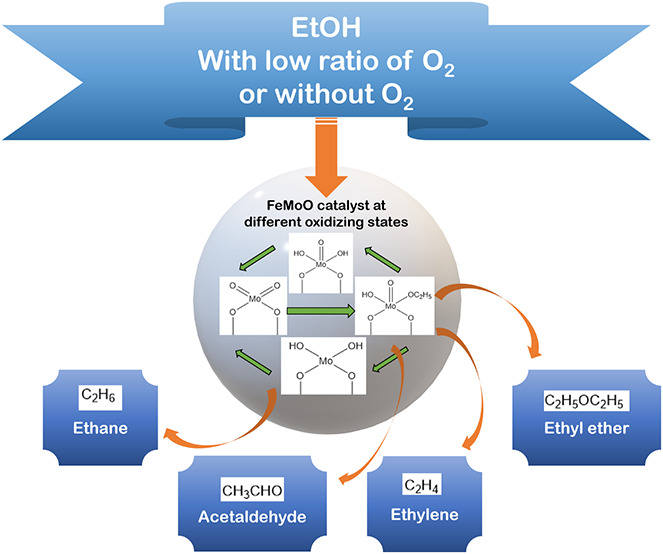

## 1 Introduction

Ethanol has been used in some countries mainly as a liquid fuel and, to a lesser extent, as a raw material for producing chemical inputs. The advent of alcohol chemistry in the 1980s motivated many groups to research efficient catalysts for various reactions. Acetaldehyde (CH_3_CHO) is a key chemical feedstock that can be produced through the catalytic conversion of bioethanol.

R1 $$\begin{array}{l} {\mathrm{CH}}_{3}{\mathrm{CH}}_{2}\mathrm{OH}\;+\;0.5O_{2}\to {\mathrm{CH}}_{3}\mathrm{CHO}\;+\;H_{2}O\\ \Delta \mathrm{Hr}=‐\mathrm{177 kJ}/\mathrm{mol} \end{array}$$

Various catalysts have been employed or investigated for the production of acetaldehyde from ethanol, either via dehydrogenation or partial oxidation ([Disp-formula eq1]), including copper-based systems (Cu/Al_2_O_3_, Cu/SiO_2_, Cu–ZnO, Cu–Cr),^1–4^ silver catalysts,^1^ vanadium- and molybdenum-based oxides,^2,5^ gold nanoparticles supported on metal oxides,^1^ as well as noble metals such as Pd, Pt, Rh, and Ir.^6,7^

Iron oxide and molybdenum oxide-based catalysts, usually used in the conversion of methanol to formaldehyde, are viable in the oxidation of ethanol to acetaldehyde.^8–11,36^ It was proposed to use a multitubular reactor with this catalyst to mitigate the exothermicity of the reaction.^12^ The conversions achieved exceeded 90% per step at a reaction temperature of 240 °C, with almost total selectivity in acetaldehyde. The O_2_/EtOH molar ratios used were between 0.76 and 6.35, i.e., within the flammability limits (NFPA-325M) for ethanol and acetaldehyde,^13^ which necessitated operation with higher air dilutions, resulting in difficulties with condensation and product separation, and was far from the maximum reactor yield. Al-Sherehy et al. proposed using an oxygen-distributed fed-reactor for oxidative dehydrogenations,^14^ ensuring that the reaction mixture is outside the flammability limits and avoiding temperature runaway along the entire length of the fixed-bed reactor. Ethanol oxidation over Fe–Mo–O catalysts has been studied for decades; however, little is known about product selectivity at low O_2_/EtOH ratios under high ethanol partial pressures. The only observation made by Iwasawa & Tanaka was that when oxygen is removed from the feed using a MoO*x*/SiO_2_ catalyst,^15^ the catalyst undergoes rapid deactivation, and the transformation of ethanol into acetaldehyde is not observed.

The present work aims to study the reactions of ethanol on a catalyst of iron and molybdenum oxides, both with and without oxygen, or with low molar O_2_/EtOH ratios, to test the viability of the distributed oxygen-fed reactor, focusing on an ethanol partial pressure above the upper flammability limit. Accordingly, this paper aims to elucidate the reaction network and derive a kinetic model that captures the main pathways under oxygen-lean operation, such as the hypothesis that ethylene acts as an H acceptor to reoxidize the site forming ethane.

## 2 Methodology and Experimental Equipment

This study used a commercial iron oxide and molybdenum catalyst with a composition of 3.2 MoO_3_·Fe_2_(MoO_4_)_3_ was used in cylindrical pellets with dimensions of 3 × 3 mm, typically used for oxidizing methanol to formaldehyde. The catalyst was characterized by X-ray fluorescence (XRF), X-ray diffraction (XRD), infrared spectrometry (FTIR), specific area, mercury intrusion pores, and TPR.

The 99.5% absolute ethanol used is supplied by Merck and is diluted with sufficient distilled and deionized water to achieve an ethanol content of 88.2% (weight basis). Nitrogen and synthetic air from White Martins were used and had 21.16% oxygen by volume.

Experiments were carried out in a Berty-type internal recycle reactor (manufactured by Autoclave Engineers, Inc., model AFPB-B16-3 3 in.); a schematic of the setup is shown in Figure 1.

**Figure 1:**
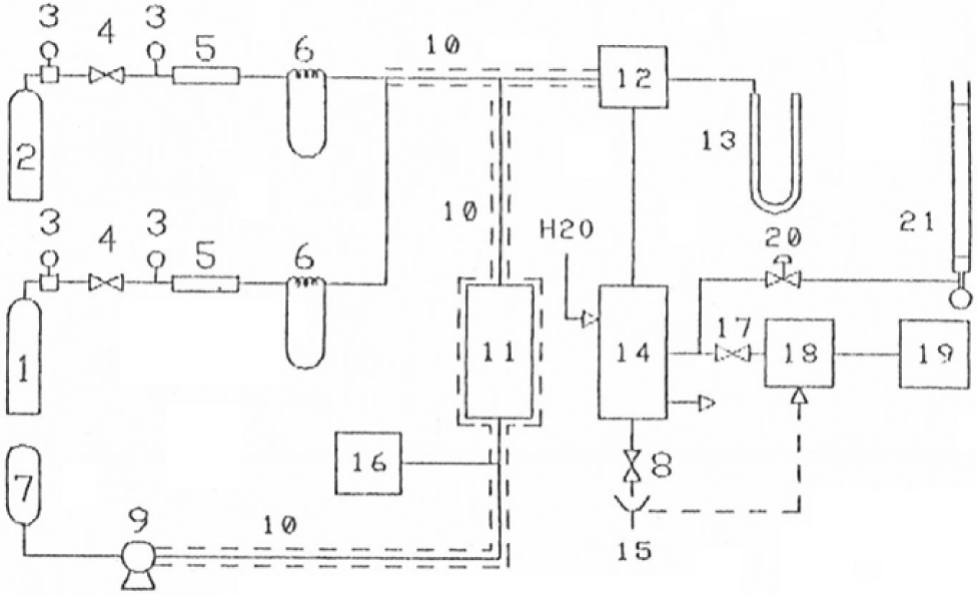
Process Schematic; Legend: 1—Synthetic air cylinder; 2—Nitrogen cylinder; 3—Pressure gauge; 4—Pressure regulating valve; 5—Tube with filling (packaging); 6—Water pressure gauge; 7—Ethanol/water mixture reservoir; 8—Ball valve; 9—Mini-dosing pump; 10—Heated tubing; 11—Vaporizer; 12—Berty reactor; 13—Mercury pressure gauge; 14—Vaporizer condenser set; 15—Output of liquid products; 16—Variable voltage drive; 17—Sampling valve; 18—Gas chromatograph; 19—Integrator; 20—Needle valve; 21—Bubble flowmeter.

The catalyst was dried at 110 °C before being placed in the reactor. The mass used in the tests was 7.2 g, packed in the basket component of the Berty Reactor, suitable for this purpose (see Figures S1 and S2 of the Supporting Information). Before reaction, the catalyst was conditioned in flowing air (at a flow rate of 500 mL/min) at the reaction temperature for 16 h.

The liquids were supplied using a positive displacement metering pump. The piping from the pump to the vaporizer-superheater was preheated utilizing electrical resistors before entering the reactor. The effluents from the reactor were subjected to a condenser separator. The flow rate of noncondensing gases was measured through a reagent flowmeter, and liquid and gaseous products were quantified by gas chromatography (GC) using a conductivity detector. To identify the substances present in both gases and liquids, GC/MS mass spectrometry was performed. The columns used were Porapack Q for gases, vapors, and condensed liquids, and 13X Molecular Sieve for light gases. In quantification, the calculation method used was normalization, using calculated factors from the literature.

The experimental data set was originally reported elsewhere.^16^ Two series of trials were conducted. The first aimed to verify the kinetics of oxidative dehydrogenation of ethanol to acetaldehyde with a low oxygen/ethanol ratio. In this condition, they worked in a region far from the flammability limit. The second series of tests aimed to verify the kinetics of dehydrogenation and other processes that occur without oxygen.

The experimental conditions used in the first series were:Ethanol concentration in water (wt
%)88.2Flow rate of the ethanol solution fed (mL/h)10.5 to 18.0Synthetic air
flow (mL/min.)17.5 to 30.0Nitrogen flow rate (mL/min)150 260Catalyst
weight (g)7.2Reactor temperature (°C)200 to 285Pressure (atm)1.03Reactor
turbine rotation (rpm)2480Space-time, W/F (g cat * h/mol EtOH)25.6 to 43.9Molar ratio O_2_/EtOH0.050 to
0.071Molar ratio EtOH/N_2_
0.40 to 0.45


The second series of experiments used the following conditions:Ethanol concentration in water (wt
%)88.2Flow rate of the ethanol solution fed (mL/h)6.0 to 60.0Nitrogen flow rate
(mL/min)80 to 300Catalyst weight (g)7.2Reactor temperature (°C)210 to 285Pressure (atm)1.03Reactor turbine rotation (rpm)2480Space-time,
W/F (g cat * h/mol EtOH)7.6 to 76.8Molar ratio O_2_/EtOH0.0Molar
ratio EtOH/N_2_
0.44 to 1.44


After the tests, the catalyst samples were also characterized for comparison with the fresh catalyst.

## 3 Results and Discussion

### 3.1 Characterization of Fresh and Used Catalyst

The fresh catalyst, subjected to X-ray fluorescence spectrometry, aimed to verify the qualitative chemical composition, revealed the presence of large proportions of iron and molybdenum, as well as traces of silicon, aluminum, calcium, titanium, copper, chlorine, and vanadium (with contents lower than 0.1% by weight). There was no variation in composition between the catalyst used and the fresh one.

Samples of the fresh catalyst, used catalyst U1 (after tests with O_2_), and used catalyst U2 (after tests without O_2_) were submitted to X-ray diffraction spectroscopy. The spectrograms obtained are shown in Figure 2a,b,c. As expected, the fresh catalyst consists of iron­(III) molybdate and molybdenum trioxide, with an approximate molar composition of 3.2 MoO_3_·Fe_2_(MoO_4_)_3_.^17,18^

**Figure 2:**
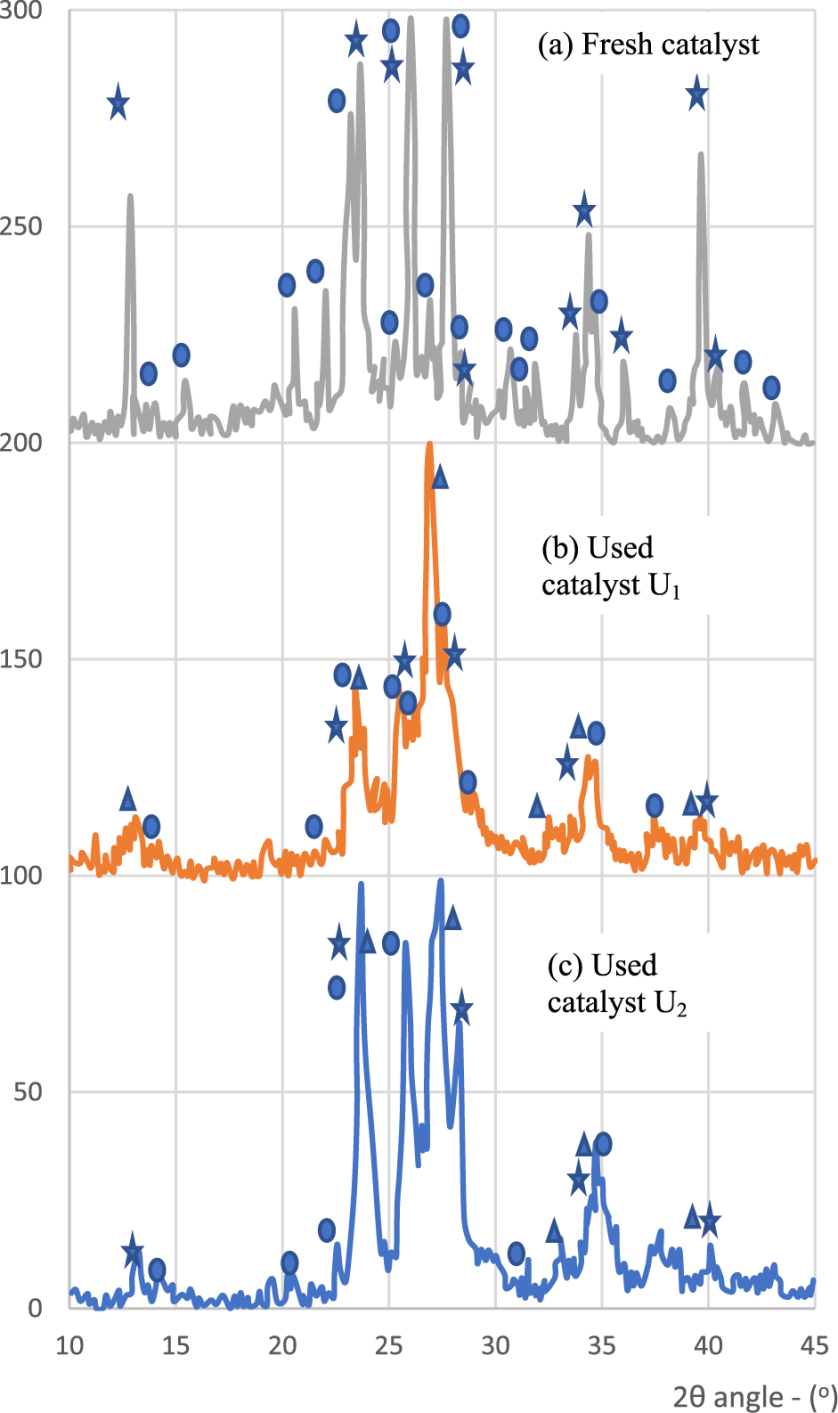
X-ray diffraction spectra.

The specific area, from fresh to used catalytic catalyst, increased by 3.3 to about 10 m^2^/gcat. There was a widening of the distribution range of macropores (200–2000 Å). It was found that pore volume increased within this distribution range from 0.21 mL/g in the fresh catalyst to 0.32 mL/g in the U1 catalyst. A calculation of the specific area in this range also shows an increase from 3.3 m^2^/g in the fresh catalyst to 6.2 m^2^/g in the U1 sample.

A possible explanation for the alterations that occurred with the catalyst is due to the crystalline changes caused by the oxidation–reduction reaction between the catalyst and the organic reagent forms. The appearance of a new phase identified in the catalysts, the β-iron­(II) molybdate, due to low or absent O_2_, causes the structural rearrangement of the catalyst, occurring with a significant variation in the volume of the unit cells.^19^

The infrared spectra of the new catalyst, using U1 and pure molybdenum oxide (MoO_3_) (prepared by calcining ammonium molybdate at 600 °C), are shown in Figure 3. Their comparison reveals the common presence of the bands at 970 cm^–1^ and 884 cm^–1^, which correspond to the groups Mo=O and Mo–O–Mo or Mo–O–Fe, respectively.^20,21^ Between the new and used catalyst (U1), there is a reduction in these bands, along with the appearance of other peaks, which could be attributed to Mo=O groups in tetrahedral coordination.^22^ In the new catalyst, the 880 cm^–1^ band appears significantly enlarged, indicating that the role of iron in this catalyst is to increase the amount of Mo–O–x bonds (where *x* = Fe or Mo).

**Figure 3:**
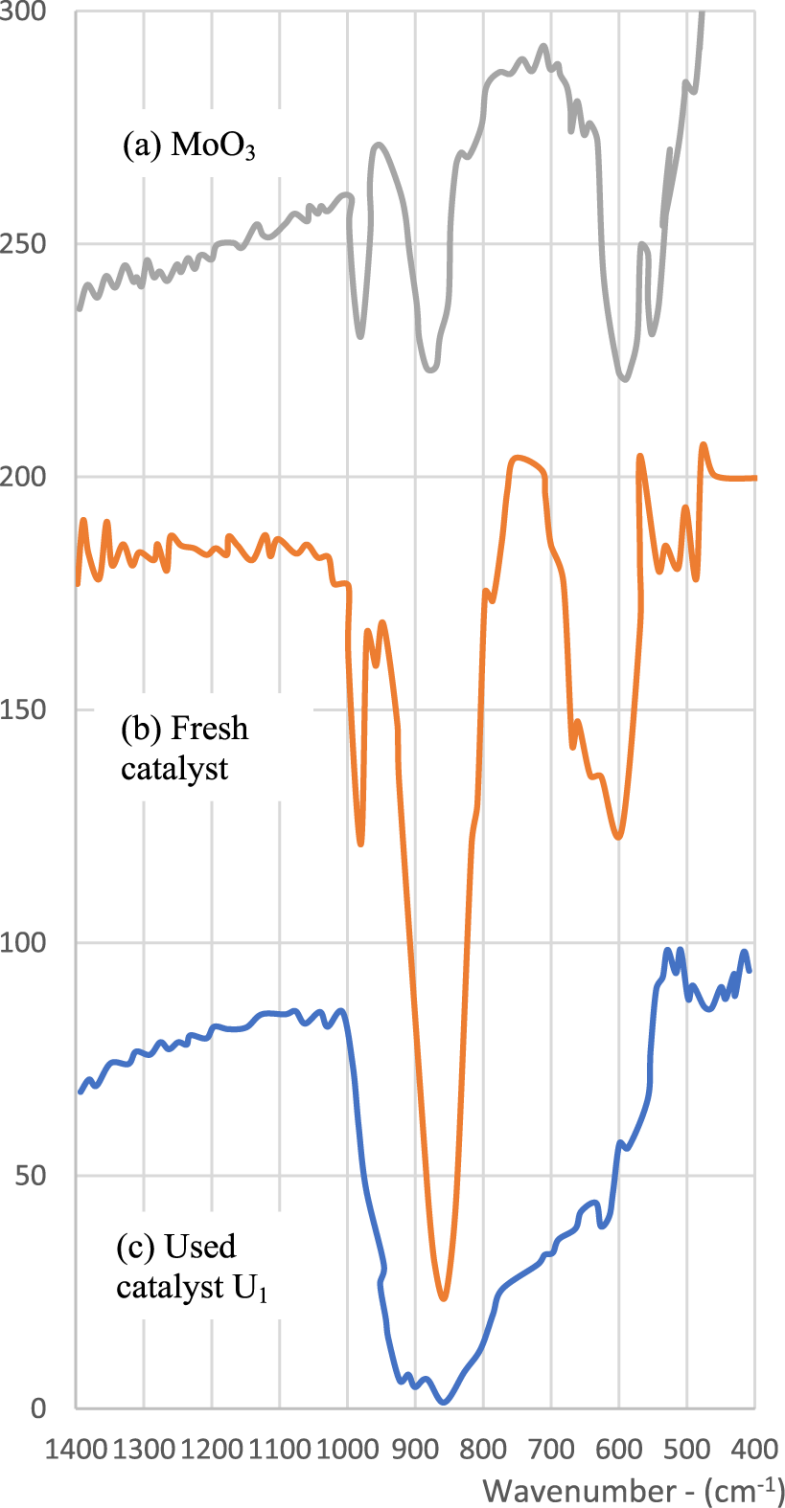
Infrared spectra.

The temperature-programmed reduction (TPR) tests were performed with a mass of approximately 100 mg, a 30 mL/min flow rate of a mixture containing 2% hydrogen in nitrogen, with a heating rate of 10 °C/min. The results are shown in Figures 4 and 5.

**Figure 4:**
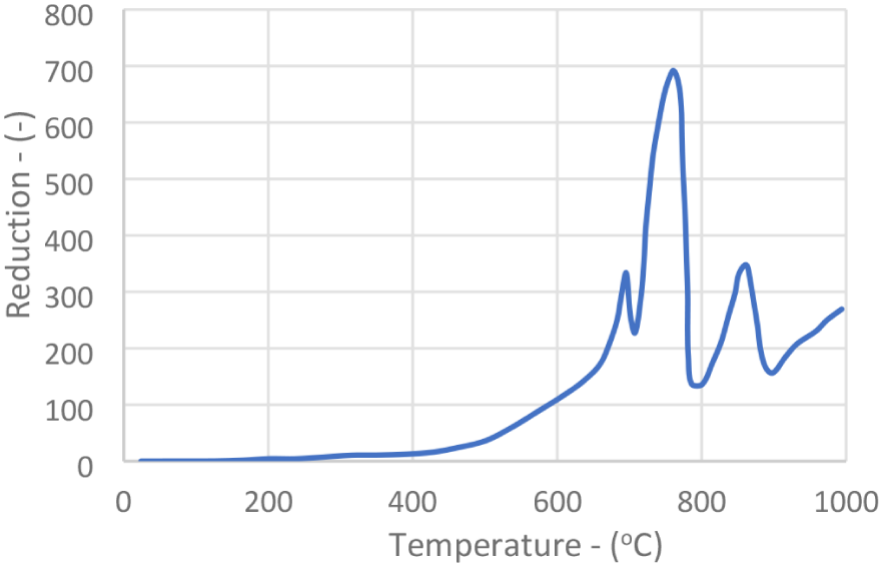
TPR of fresh catalyst.

**Figure 5:**
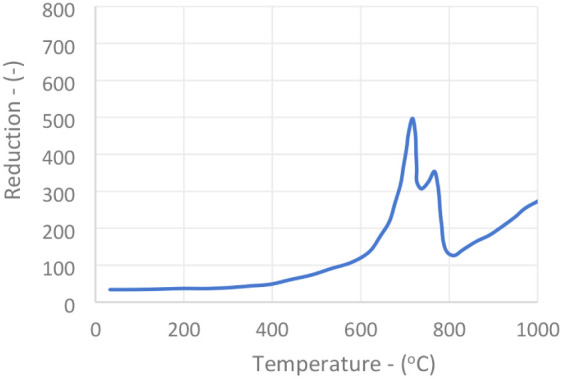
TPR of used catalyst U1.

It can be noted that an essential difference between the new and used catalyst is the existence of a peak with a maximum of 850 °C. This peak is absent in molybdenum oxide samples. It, therefore, must refer to some characteristic of the iron­(III) molybdate, a constituent of the new catalyst that ceases to exist in the used U1 sample. Another difference observed between the New and Used catalyst (U1) is the relatively sharp reduction of the peak at 735 °C and the increase at 700 °C. One possible explanation for this last peak is that the MoO_3_ secreted by the reduction (inverse of the [Disp-formula eq2] reaction) of iron­(III) molybdate may be more easily reducible than that coordinated with iron. This MoO_3_ may be the species seen in infrared light with a wavelength of around 920 cm^–1^.

R2 $${\beta \mathrm{‐FeMoO}}_{4}{\;+\;\mathrm{MoO}}_{3}{\;+\;1/\mathrm{2 O}}_{2}\Leftrightarrow \mathrm{Fe2}{\left({\mathrm{MoO}}_{4}\right)}_{3}$$

### 3.2 Product Spectrum—Qualitative Mechanistic Inferences

Mass spectrometry (by GC/MS) of the reaction products revealed the presence of carbon dioxide, ethylene, ethane, water, propylene, acetaldehyde, butene, butadiene, butane, ethanol, ethyl ether, methylbutene, and ethyl acetate in the gas phase and water, acetaldehyde, ethanol, ethyl ether, 2,3-epoxybutane, ethyl acetate, crotonaldehyde, 2-methyl-3-propanol, and ethyl *n*-butanoate in the liquid phase. The catalyst’s acid–base character induces the formation of substances originating from both basic catalysis, such as acetaldehyde, and acid catalysis, including ethylene and ethyl ether. This characteristic indicates that the catalyst in this system, under the studied conditions, is not very selective.

From the species present in the reactive system and based on the possible reactions presented in the literature,^23,24,37–40^ it was concluded that the most probable reaction network should involve the following reactions:

R3 Ethanol→ethylene+water

R4 2 Ethanol→ethyl ether+water

R5 Ethyl ether→2 ethylene+water

R6 Ethyl ether→ethane+acetaldehyde

R7 Ethanol+ethylene→ethane+acetaldehyde

R8 2 Acetaldehyde→ethyl acetate

R9 2 Acetaldehyde→other derivatives

Of note is the appearance of ethyl acetate, without the presence of acetic acid and hemiacetal, indicating that it can be produced by a Tishchenko reaction,^23,24^ represented by the [Disp-formula eq8] reaction.

The most widely used kinetic models that consider aspects of the behavior of substances adsorbed on the surface of the catalyst are those of the LHHW (Langmuir–Hinshelwood-Hougen-Watson) type. However, given the number of reactions that must be considered and the high number of parameters, these types of models were discarded a priori.

### 3.3 Proposed Reaction Network

Approximately 20 models were tested, with variations in site type and reaction order. Since some tested kinetic models did not accurately represent the data and the confidence intervals of the parameters were too large, these models were discarded.

The proposition of a consistent reaction network that accurately represents the data arose from the analysis of Figure 6. This figure shows the variation between the partial pressure of ethane and acetaldehyde. The presence of oxygen causes a displacement of the curve as if there were an inhibiting effect on the appearance of ethane. This suggests that ethylene may act as a hydrogen acceptor, instead of oxygen, to reoxidize reduced surface sites, producing ethane as a possible product of the catalyst’s reoxidation.

**Figure 6:**
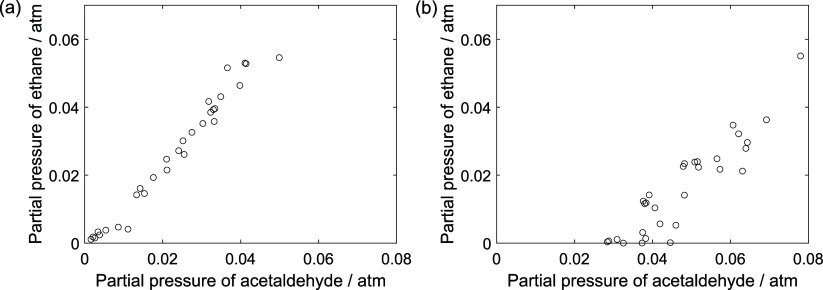
Partial pressure of ethane as a function of partial pressure of acetaldehyde: (a) experiments in the absence of oxygen; (b) experiments in the presence of oxygen; ○, experimental data.

A mechanistic picture consistent with the observations combines a Mars–van Krevelen-type redox cycle with surface ethoxide intermediates:

- Formation of surface ethoxide on oxidized sites (EtOH + CatO → EtO–Cat + HO*).
- Competing consumption of ethoxide to ethylene + H_2_O (dehydration), to ethyl ether + H_2_O (condensation), or to acetaldehyde + reduced site (dehydrogenation).
- Reoxidation of reduced sites by ethylene as hydrogen acceptor, yielding ethane (in O_2_-free cases), and by O_2_ when cofed (forming H_2_O).

This framework is aligned with prior reports on alcohol oxidation over metal oxides and provides a compact basis for kinetics modeling in Section 3.4.

The reaction network that could result from this fact is the MVK type (Mars & Van Krevelen).^25^ But in the present study, the oxygen is replaced by ethylene according to the [Disp-formula eq10] and 11 reactions, considering its occurrence on oxidized or reduced sites.

R10 Oxidized cat.+ethanol⇒acetaldehyde+reduced
cat

R11 Reduced cat.+ethylene⇒ethane+oxidized cat.

The developed reaction network also considers information about surface reactions, formation of intermediates, and desorption of products. If the catalyst in the oxidized state is responsible for the reaction, and since alcohols could adsorb on the surface of the catalysts, forming ethoxide groups independent of the acidic or basic nature of the catalyst,^26,27^ it was assumed that this ethoxide would be an intermediate common to the three ethanol reactions ([Disp-formula eq12]).

The subsequent reactions of this intermediate would be the formation of ethyl ether, acetaldehyde, and ethylene. The formation of ethylene would involve the loss of beta hydrogen with the desorption of ethylene ([Disp-formula eq13]).^28^

The ether would be formed due to the reaction of two adjacent ethoxide groups, which would be more likely to form with high contact times when little oxygen would be available on the catalyst’s surface ([Disp-formula eq14]).^29^ In the present case, it is believed that, in addition to the absence of oxygen, the high partial pressure of ethanol also contributes to forming a large concentration of ethoxide groups and, consequently, of ethyl ether.

Acetaldehyde would be formed by subtracting one hydrogen atom (dehydrogenation) from the carbon adjacent to oxygen in the adsorbed ethoxide group ([Disp-formula eq15]). According to the literature, this would determine the speed of the reaction.^15,27,30,31^

Ethane would form by the possible transfer of hydrogen from adjacent hydroxyls to the ethylene present in the gas. These hydroxyls bonded to molybdenum atoms in a reduced state ([Disp-formula eq16]). This approach differs from the one proposed by Nakamura et al.^40^ with V_2_O_3_·MoO_2_ catalyst, which considered a simultaneous generation of ethane and acetaldehyde instead of an ethoxide intermediate that generates ethylene as one of initial steps.

Also, in the presence of oxygen, the reduced site of the catalyst can be oxidized, leading to the generation of water ([Disp-formula eq17]).^15^

The water would be formed by the dehydration of two adjacent hydroxyls belonging to molybdenum atoms in the most oxidized state in the [Disp-formula eq13] and 14 steps. However, for the sake of simplification and also because it does not lead to significant improvement, this reaction has not been considered separately. A representation of the reaction network can be seen in Figure 7. The consideration of only the substances present in the proposed reaction and the neglect of other substances previously mentioned (propylene, butene, butadiene, butane, methylbutene, ethyl acetate, 2,3-epoxybutane, crotonaldehyde, 2-methyl-3-propanol, and ethyl *n*-butanoate) can be justified by their low quantities compared to the other substances, leading to average errors of 5.8%, 5.8%, and 5.1% for the atoms of carbon, oxygen, and hydrogen, respectively, after performing atomic balances between the feed and exit substances. This reduces the mathematical complexity associated with the reaction network.

**Figure 7:**
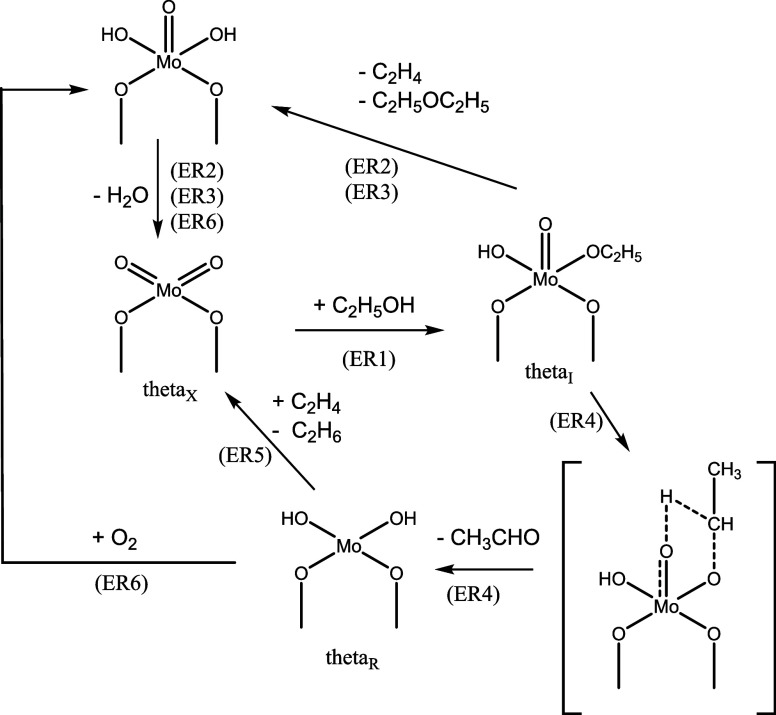
Representation of the proposed reaction network.

Thus, the complete scheme would be given by the steps:

ER1 Ethanol+oxidized site→intermediate site

ER2 Intermediate site→ethylene+water+oxidized side

ER3 2 Intermediate site→ethyl ether+water+2 oxidized site

ER4 Intermediate site→acetaldehyde+reduced site

ER5 Ethylene+reduced site→ethane+oxidized site

ER6 Oxygen+2 reduced site→2 water+2 oxidized
site

### 3.4 Formulation of the Mathematical Model

The transformation of ethanol over the iron–molybdenum oxide catalyst in the absence of oxygen, given by steps [Disp-formula eq12] to 17, can then be expressed by the following model. Eq 18 sums to unity the fractions of the considered states of catalyst sites.

1 $$\theta_{X}+\theta_{R}+\theta_{I}=1$$

where θ is the fraction of the site, and the subscripts X, R, and I refer to oxidized, reduced, and intermediate sites, respectively.

The rates of each step were described by an elementary power-law model:

2 $$r_{1}=k_{1}\times P_{EtOH}\times \theta_{X}$$

3 $$r_{2}=k_{2}\times \theta_{I}$$

4 $$r_{3}=k_{3}\times {\theta_{I}}^{2}$$

5 $$r_{4}=k_{4}\times \theta_{I}$$

6 $$r_{5}=k_{5}\times \theta_{R}\times P_{ETHYLENE}$$

7 $$r_{6}=k_{6}\times \theta_{R}^{2}\times P_{OXYGEN}$$

where *r* is the reaction rate related to each reaction, *k* is the kinetic constant, and *P* is the partial pressure.

The Arrhenius equation was included to account for the temperature effects:

8 $$k_{i}=k_{i0}\times \exp\left[\frac{E_{ai}}{R_{g}}\left(\frac{1}{T_{m}}-\frac{1}{T}\right)\right]$$

where *k*_0_ is the kinetic constant at the reference temperature *T*_m_, *E*_a_ is the energy of activation, *R*_g_ is the universal gas constant, and *T* is the system temperature.

The reaction rates (R) related to the surface sites wouldbe

9 $$R_{\theta_{OXID}}=-r_{1}+r_{2}+2\times r_{3}+r_{5}+2\times r_{6}$$

10 $$R_{\theta_{RED}}=r_{4}-r_{5}-2\times r_{6}$$

11 $$R_{\theta_{INT}}=r_{1}-r_{2}-2\times r_{3}-r_{4}$$

Where using the Pseudo Steady State Hypothesis (PSSH):^32^

12 $$R_{\theta_{X}}=R_{\theta_{R}}=R_{\theta_{I}}=0$$

Therefore, eqs 18 to ([Disp-formula eq29]) yield:

13 $$a\theta_{R}^{4}+b\theta_{R}^{3}+c\theta_{R}^{2}+d\theta_{R}+e=0$$

14 $$\theta_{I}=\frac{k_{5}\times P_{ETHYLENE}\times \theta_{R}+2\times k_{6}\times P_{OXYGEN}\times \theta_{R}^{2}}{k_{4}}$$

15 $$\theta_{X}=\frac{\left(k_{2}+k_{4}\right)\times \theta_{I}+2\times k_{3}\times \theta_{I}^{2}}{k_{1}\times P_{EtOH}}$$

Where:

16 $$a=8\times k_{3}\times k_{6}^{2}\times P_{\mathrm{OXYGEN}}^{2}$$

17 $$b=8\times k_{3}\times k_{5}\times k_{6}\times P_{\mathrm{ETHYLENE}}\times P_{\mathrm{OXYGEN}}$$

18 $$c=2\times k_{4}\times k_{6}\times \left(k_{2}+k_{4}+k_{1}\times P_{EtOH}\right)\times P_{\mathrm{OXYGEN}}+2\times k_{3}\times k_{5}^{2}\times P_{ETHYLENE}^{2}$$

19 $$d=k_{4}\times k_{5}\times \left(k_{2}+k_{4}+k_{1}\times P_{EtOH}\right)\times P_{\mathrm{ETHYLENE}}+k_{1}\times k_{4}^{2}\times P_{EtOH}$$

20 $$e=-k_{1}\times k_{4}^{2}\times P_{EtOH}$$

From the experimental data, values of the formation or consumption rates of each species are obtained by eq 38), based on the design equation for the reactor, which was assumed to be a continuous stirred tank reactor (CSTR) with catalyst (fluidized bed) in steady state:

21 $$R_{j,exp}=\frac{F_{j}-F_{j0}}{W}$$

where *F*_j0_ and *F*_j_ are the feed and exit molar flow rates of the j substance, W is the catalyst mass, and the subscript “exp” refers to experimental values.

The value of the reaction rate estimated by the model (*R*_mod_) is the result of the various steps of the proposed model, based on the stoichiometric coefficients (α). So:

22 $$R_{j,mod}=\overset{m}{\underset{k=1}{\sum }}\propto_{j\times k}\times r_{k}\;,\;\left(j=1,n\right)$$

and:

23 $$R_{EtOH}=-r_{1}$$

24 $$R_{H_{2}O}=r_{2}+r_{3}+2\times r_{6}$$

25 $$R_{\mathrm{ETHYLENE}}=r_{2}-r_{5}$$

26 $$R_{\mathrm{ETHER}}=r_{3}$$

27 $$R_{AcH}=r_{4}$$

28 $$R_{\mathrm{ETHANE}}=r_{5}$$

29 $$R_{\mathrm{OXYGEN}}=-r_{6}$$

As a preliminary evaluation, Figures 8–14 present the experimental values of the reaction rate as a function of the ethanol feed pressure, while Figures 15–21 show the experimental values of pressure as a function of temperature. The reaction rate of ethanol tended to increase (in absolute value) for higher ethanol feed pressures, while the reaction rate of oxygen showed a decreasing tendency (in absolute value). This suggests an intensified consumption of oxygen when ethanol is added in higher amounts. The reaction rate of ethanol also decreased in the presence of oxygen, which was accompanied by a higher consumption of ethanol, resulting in lower partial pressures of ethanol at the exit. Although it was not possible to infer the tendency of the reaction rates for the products with respect to the ethanol feed pressure, the presence of oxygen at least had a significant effect on the decrease of the ethane reaction rate, which can be attributed to the preference of the reduced sites of the catalyst to undergo to reaction 17 instead of [Disp-formula eq16]. Conversely, all products exhibited an increasing tendency in their partial pressures as the temperature increased, while ethanol showed a decreasing tendency, which is expected due to the increase in the kinetic constant with temperature. However, the intensified increase in the partial pressures of acetaldehyde and ethyl ether is noteworthy in the presence of oxygen, compared to an environment without oxygen.

**Figure 8:**
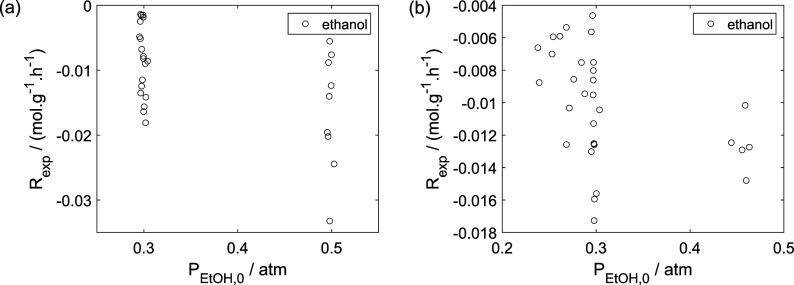
Experimental reaction rate of ethanol as a function of the ethanol feed partial pressure: (a) experiments in the absence of oxygen; (b) experiments in the presence of oxygen; ○, experimental data.

**9:**
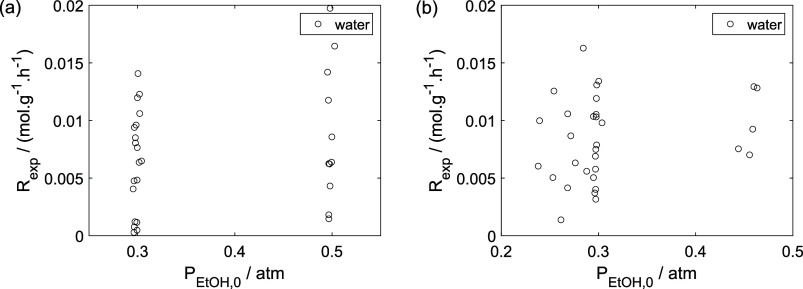
Experimental reaction rate of water as a function of the ethanol feed partial pressure: (a) experiments in the absence of oxygen; (b) experiments in the presence of oxygen; ○, experimental data.

**10:**
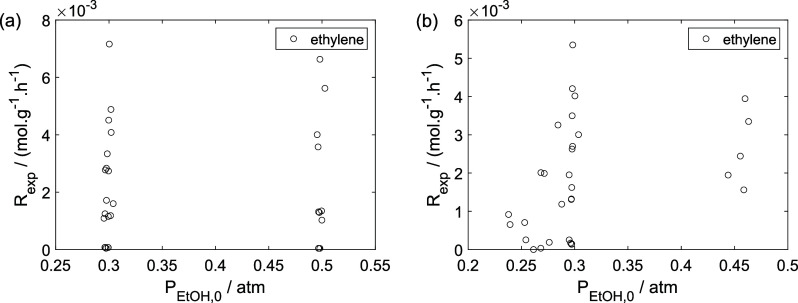
Experimental reaction rate of ethylene as a function of the ethanol feed partial pressure: (a) experiments in the absence of oxygen; (b) experiments in the presence of oxygen; ○, experimental data.

**11:**
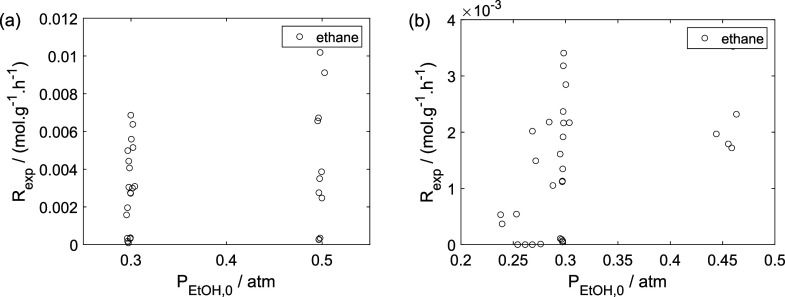
Experimental reaction rate of ethane as a function of the ethane feed partial pressure: (a) experiments in the absence of oxygen; (b) experiments in the presence of oxygen; ○, experimental data.

**12:**
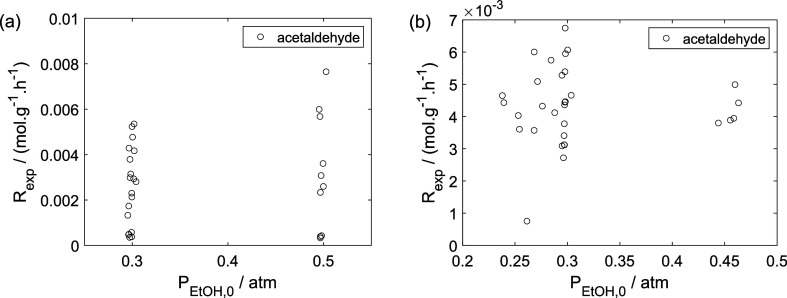
Experimental reaction rate of acetaldehyde as a function of the ethanol feed partial pressure: (a) experiments in the absence of oxygen; (b) experiments in the presence of oxygen; ○, experimental data.

**13:**
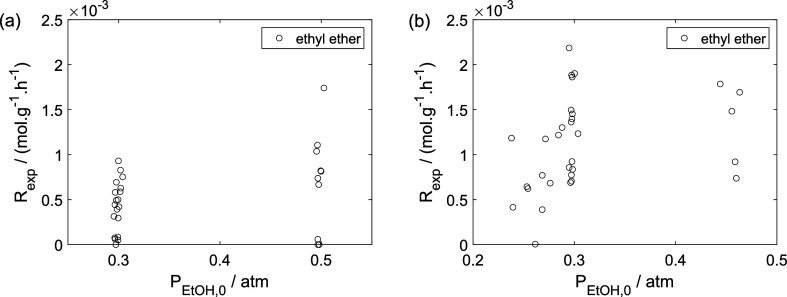
Experimental reaction rate of ethyl ether as a function of the ethanol feed partial pressure: (a) experiments in the absence of oxygen; (b) experiments in the presence of oxygen; ○, experimental data.

**Figure 14:**
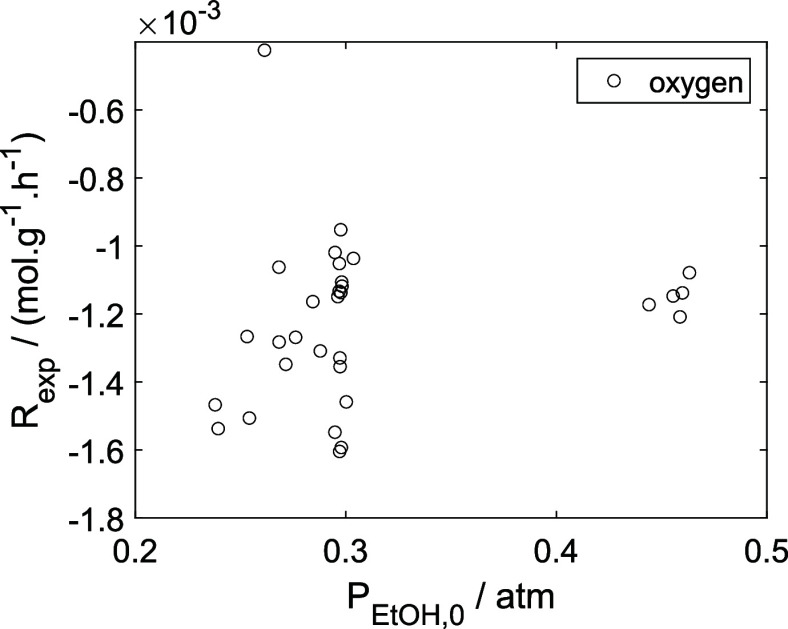
Experimental reaction rate of oxygen as a function of the ethanol feed partial pressure in the experiments in the presence of oxygen; ○, experimental data.

**Figure 15:**
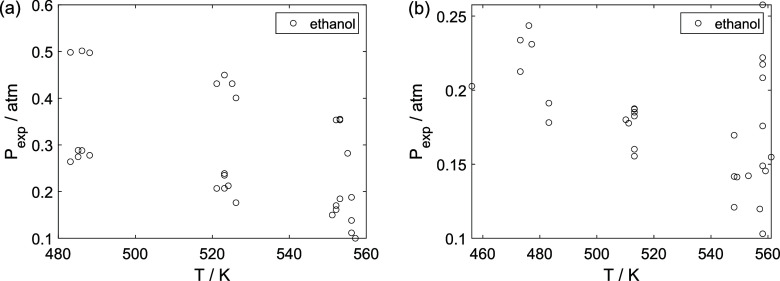
Experimental partial pressure of ethanol as a function of the temperature: (a) experiments in the absence of oxygen; (b) experiments in the presence of oxygen; ○, experimental data.

**16:**
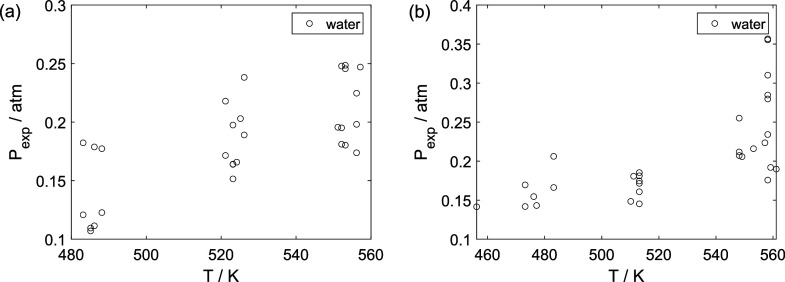
Experimental partial pressure of water as a function of the temperature: (a) experiments in the absence of oxygen; (b) experiments in the presence of oxygen; ○, experimental data.

**17:**
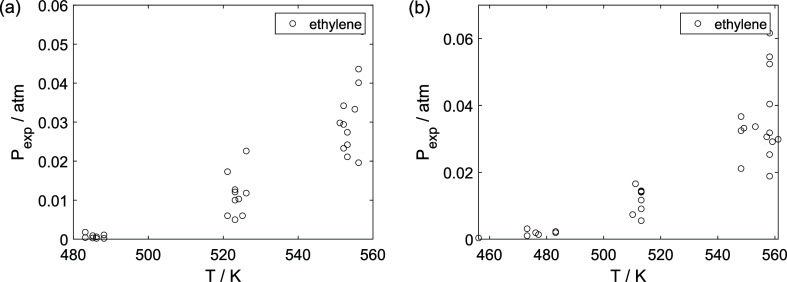
Experimental partial pressure of ethylene as a function of the temperature: (a) experiments in the absence of oxygen; (b) experiments in the presence of oxygen; ○, experimental data.

**18:**
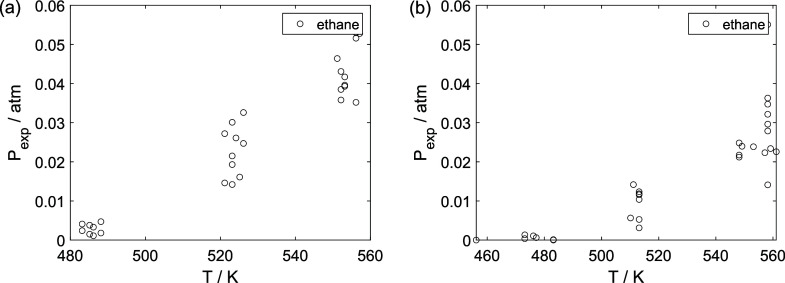
Experimental partial pressure of ethane as a function of the temperature: (a) experiments in the absence of oxygen; (b) experiments in the presence of oxygen; ○, experimental data.

**19:**
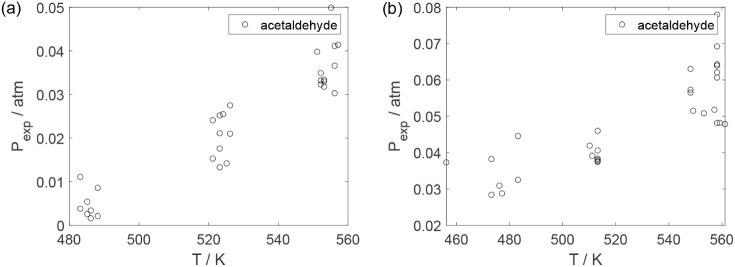
Experimental partial pressure of acetaldehyde as a function of the temperature: (a) experiments in the absence of oxygen; (b) experiments in the presence of oxygen; ○, experimental data.

**20:**
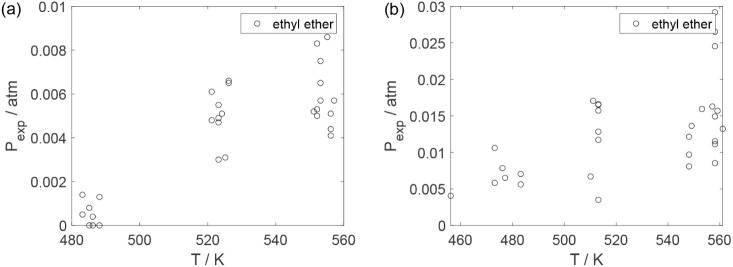
Experimental partial pressure of ethyl ether as a function of the temperature: (a) experiments in the absence of oxygen; (b) experiments in the presence of oxygen; ○, experimental data.

**Figure 21:**
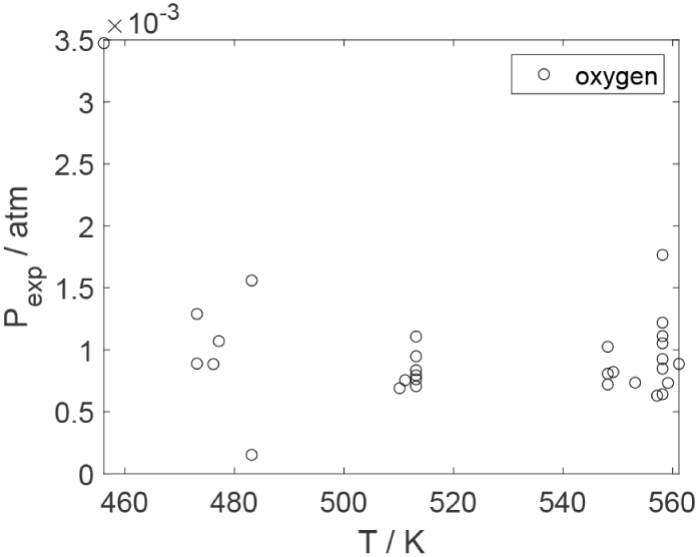
Experimental partial pressure of oxygen as a function of the temperature in the experiments in the presence of oxygen; ○, experimental data.

### 3.5 Kinetic Model Adjustment, Parameter Estimation, and Parity Plots

Given the number of reactions and potential site types, full LHHW treatments were screened and discarded due to parameter nonidentifiability and poor confidence bounds. We therefore formulated a reduced model reflecting the steps above and fitted it by maximum likelihood using Marquardt’s algorithm,^33,34^ using the software Matlab (v.2021b), with the command “lsqnonlin”. Estimated kinetic constants at the reference temperature (*k*_*i*_0 at *T*_m_ = 531.5 K) and activation parameters (*E*a_*i*_/R) are summarized in Table 1, jointly with the 95% confidence intervals. Parity plots (Figures 22–28) compare experimental and model-predicted rates for ethanol, water, ethylene, ethane, acetaldehyde, ethyl ether, and oxygen; most points fall near *y* = *x* within ±25% envelopes. Additionally, Figures 29–35 present parity plots for the partial pressures of all substances.

**Table 1:** Values of the Estimated Parameters

Reaction	*k*_*i*o_ at *T*_m_ = 531.5 K	*E*_a*i*_/R (K)
r1	(1.278 ± 0.149) × 10^–1^ mol·g^–1^·h^–1^·atm^–1^	(2.557 ± 0.105) × 10^3^
r2	(1.294 ± 0.162) × 10^–2^ mol·g^–1^·h^–1^	(6.093 ± 1.071) × 10^3^
r3	(4.035 ± 0.936) × 10^–3^ mol·g^–1^·h^–1^	(3.051 ± 1.979) × 10^3^
r4	(8.801 ± 1.061) × 10^–3^ mol·g^–1^·h^–1^	(3.402 ± 1.018) × 10^3^
r5	(2.034 ± 0.396) × 10^0^ mol·g^–1^·h^–1^·atm^–1^	(8.405 ± 2.346) × 10^3^
r6	(1.340 ± 0.417) × 10^3^ mol·g^–1^·h^–1^·atm^–1^	(1.190 ± 0.416) × 10^4^

**Figure 22:**
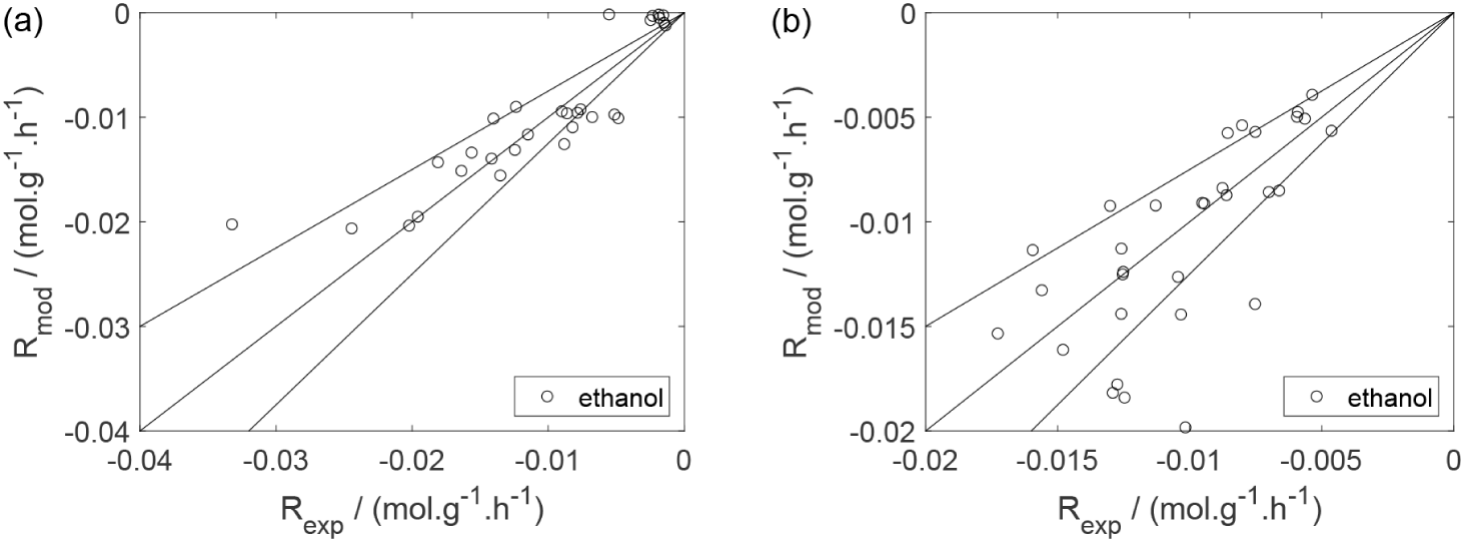
Parity plot for the reaction rate for ethanol: (a) experiments in the absence of oxygen; (b) experiments in the presence of oxygen; ○, experimental data; lines represent *y* = *x* and deviations of ±25%.

**23:**
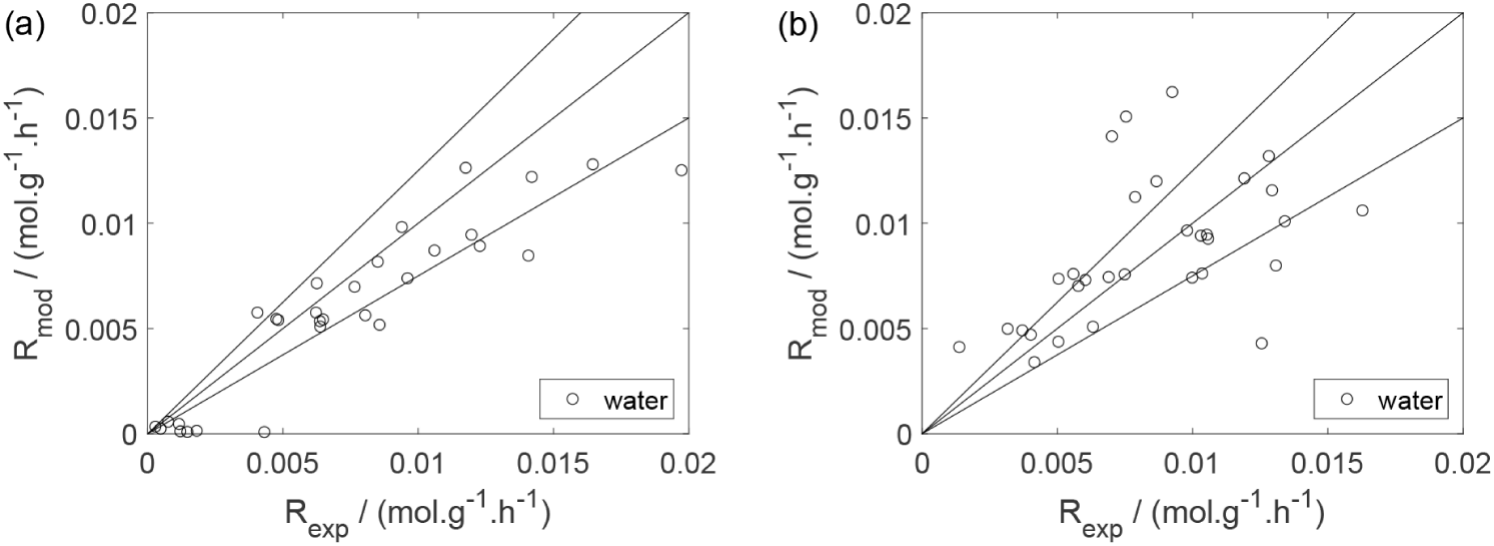
Parity plot for the reaction rate for water: (a) experiments in the absence of oxygen; (b) experiments in the presence of oxygen; ○, experimental data; lines represent *y* = *x* and deviations of ±25%.

**24:**
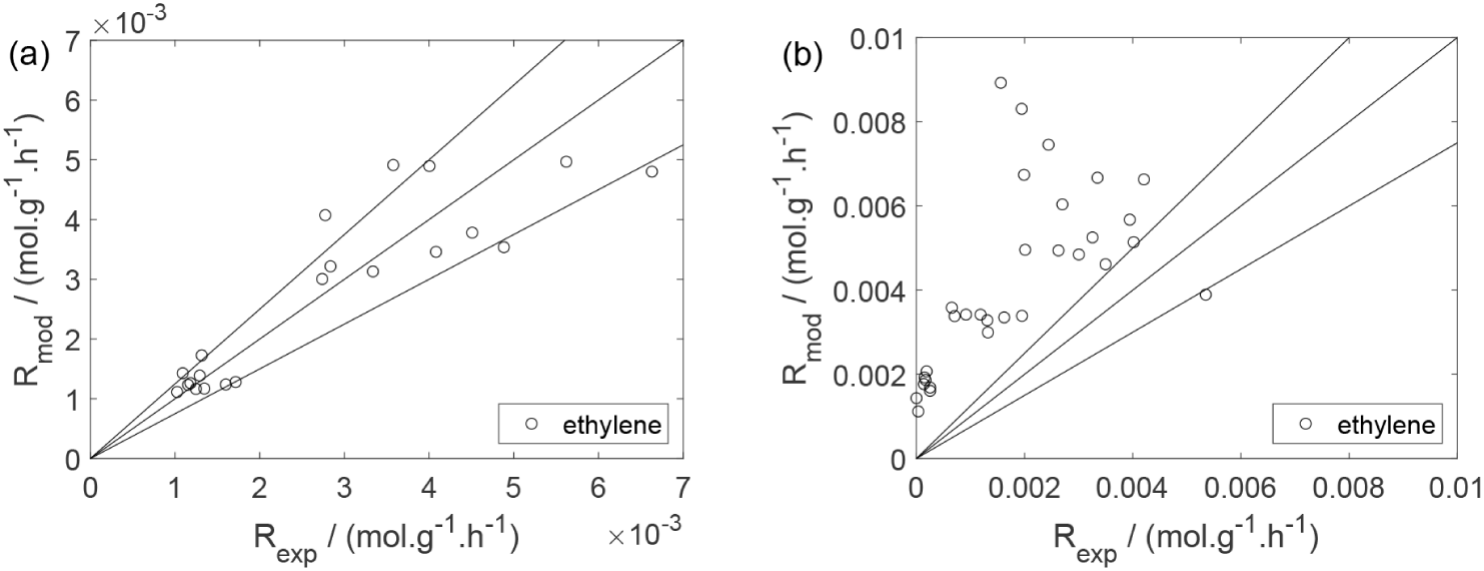
Parity plot for the reaction rate for ethylene: (a) experiments in the absence of oxygen; (b) experiments in the presence of oxygen; ○, experimental data; lines represent *y* = *x* and deviations of ±25%.

**25:**
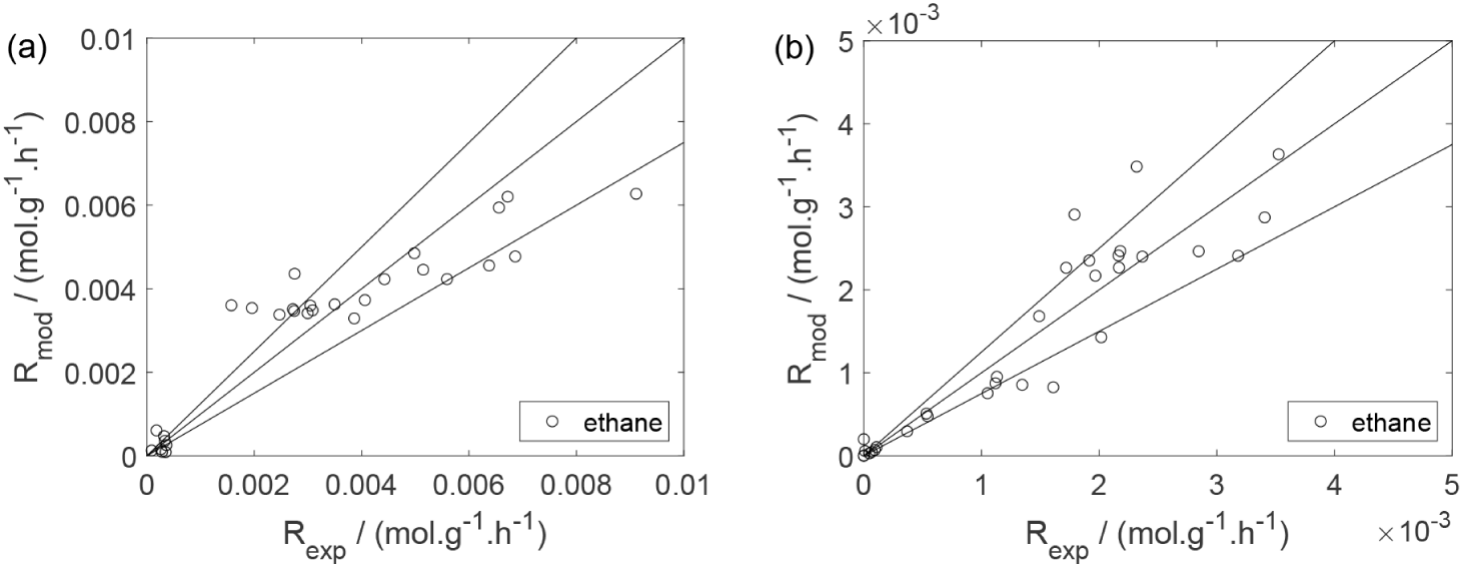
Parity plot for the reaction rate for ethane: (a) experiments in the absence of oxygen; (b) experiments in the presence of oxygen; ○, experimental data; lines represent *y* = *x* and deviations of ±25%.

**26:**
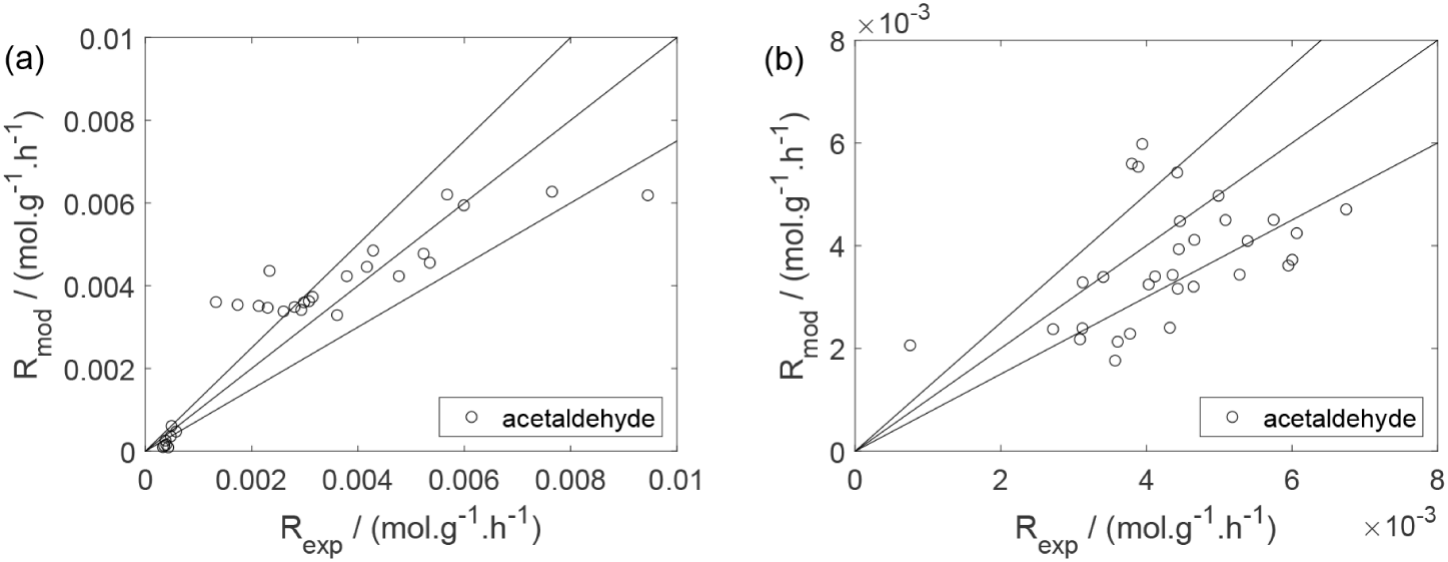
Parity plot for the reaction rate for acetaldehyde: (a) experiments in the absence of oxygen; (b) experiments in the presence of oxygen; ○, experimental data; lines represent *y* = *x* and deviations of ±25%.

**27:**
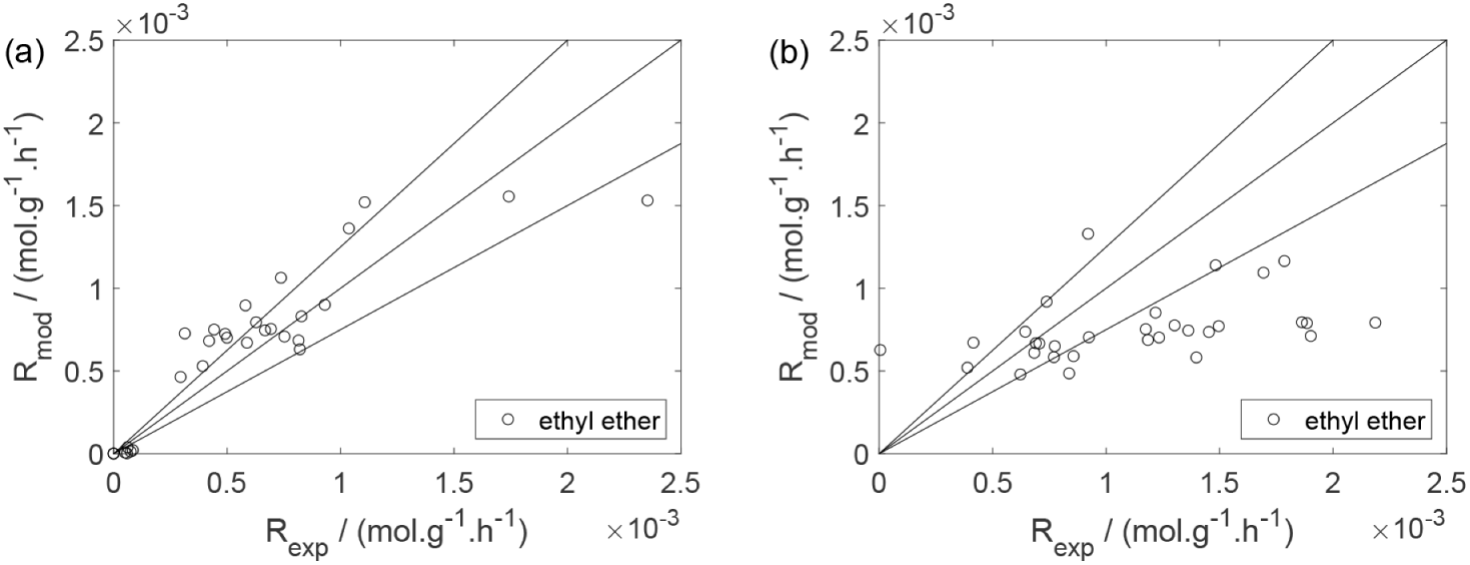
Parity plot for the reaction rate for ethyl ether: (a) experiments in the absence of oxygen; (b) experiments in the presence of oxygen; ○, experimental data; lines represent *y* = *x* and deviations of ±25%.

**Figure 28:**
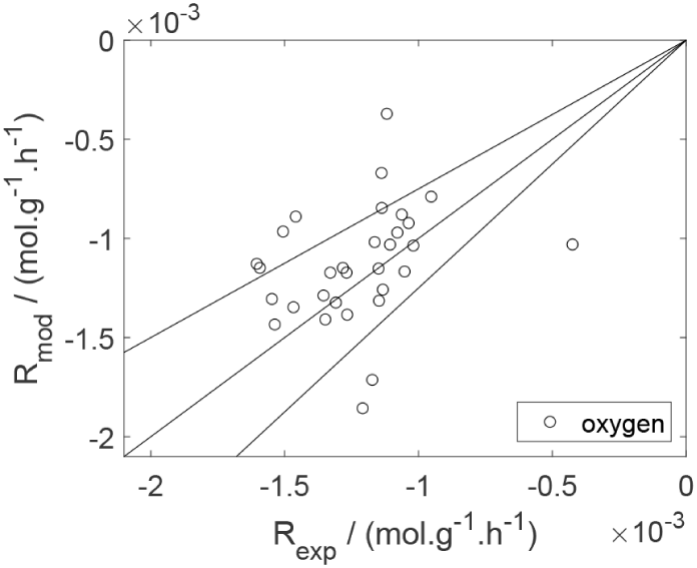
Parity plot for the reaction rate for oxygen in the experiments in the presence of oxygen; ○, experimental data; lines represent *y* = *x* and deviations of ±25%.

**Figure 29:**
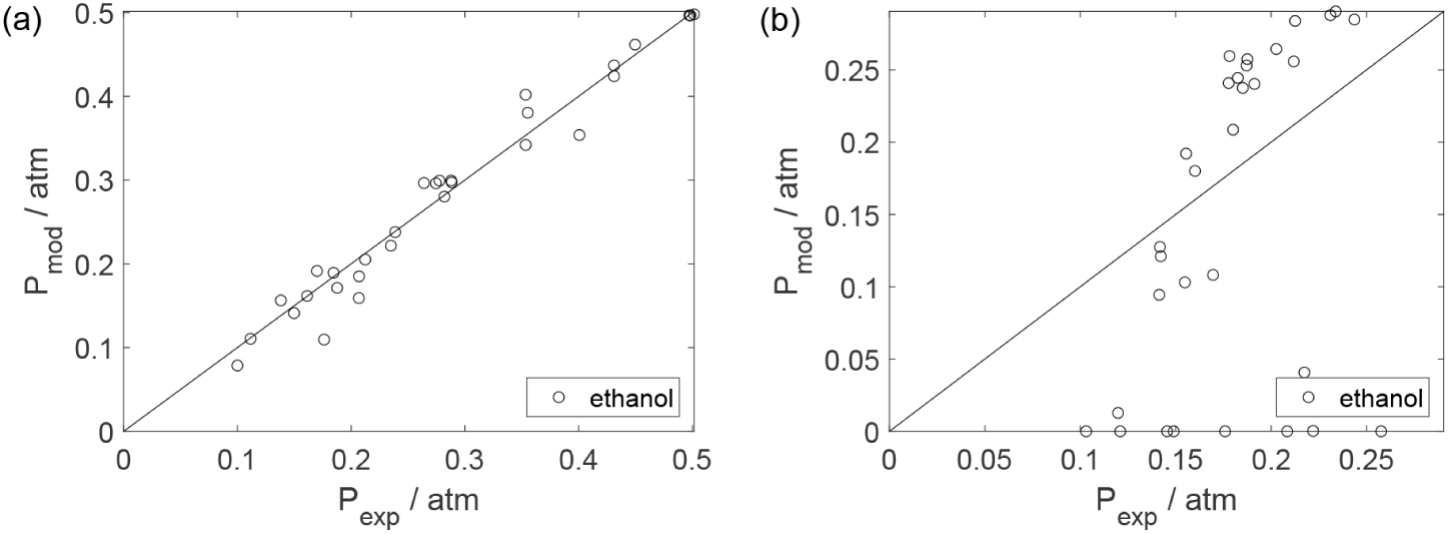
Parity plot for the partial pressure of ethanol: (a) experiments in the absence of oxygen; (b) experiments in the presence of oxygen; ○, experimental data; lines represent *y* = *x*.

**30:**
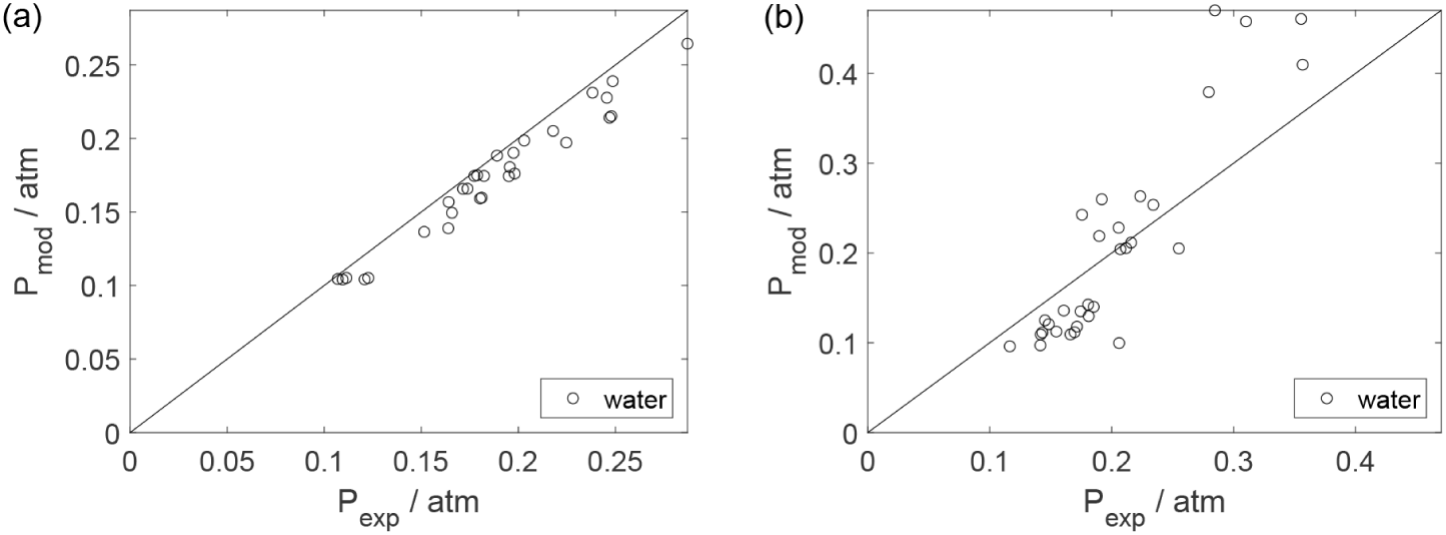
Parity plot for the partial pressure of water: (a) experiments in the absence of oxygen; (b) experiments in the presence of oxygen; ○, experimental data; lines represent *y* = *x*.

**31:**
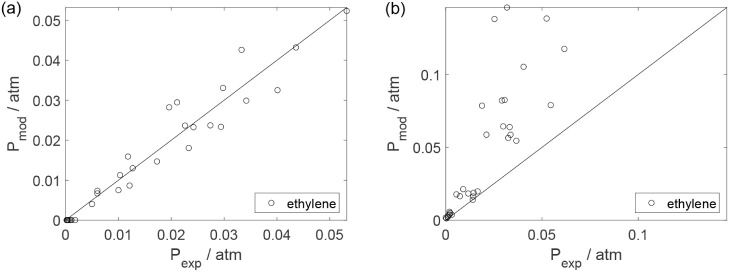
Parity plot for the partial pressure of ethylene: (a) experiments in the absence of oxygen; (b) experiments in the presence of oxygen; ○, experimental data; lines represent *y* = *x*.

**32:**
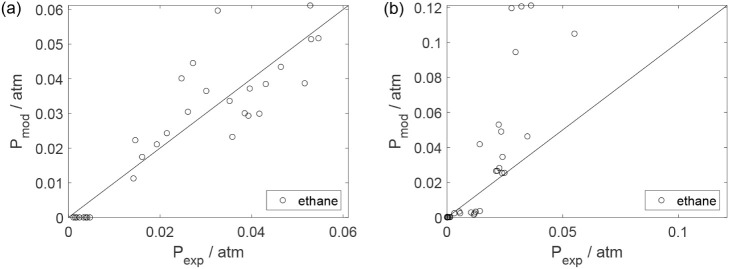
Parity plot for the partial pressure of ethane: (a) experiments in the absence of oxygen; (b) experiments in the presence of oxygen; ○, experimental data; lines represent *y* = *x*.

**33:**
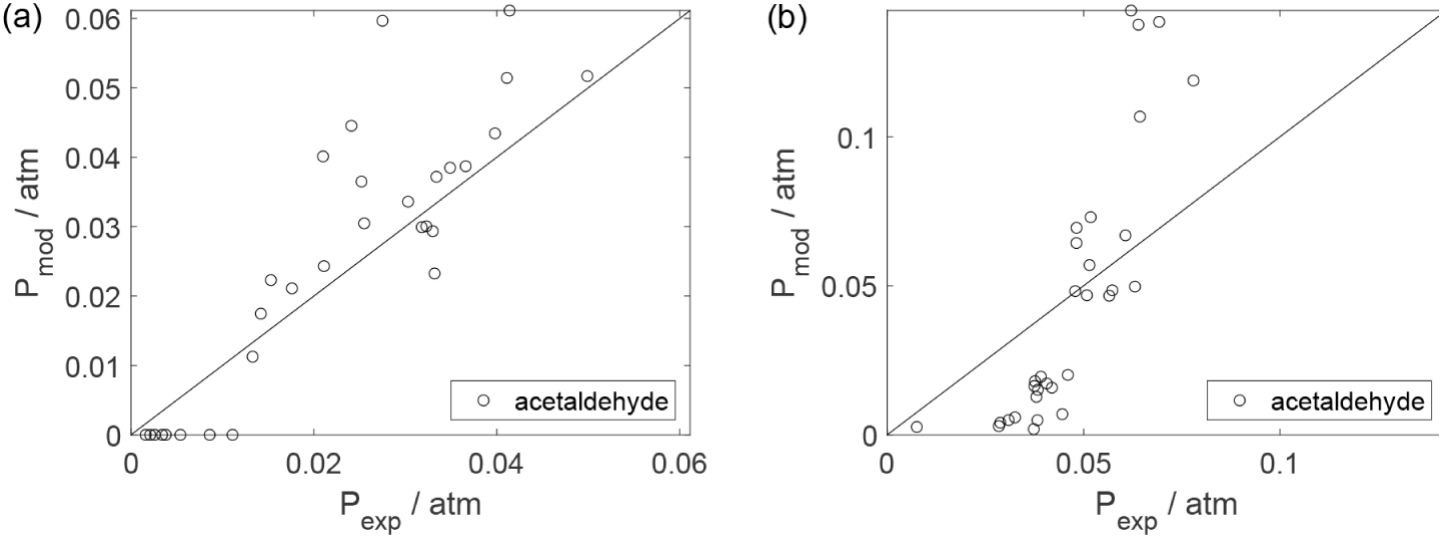
Parity plot for the partial pressure of acetaldehyde: (a) experiments in the absence of oxygen; (b) experiments in the presence of oxygen; ○, experimental data; lines represent *y* = *x*.

**34:**
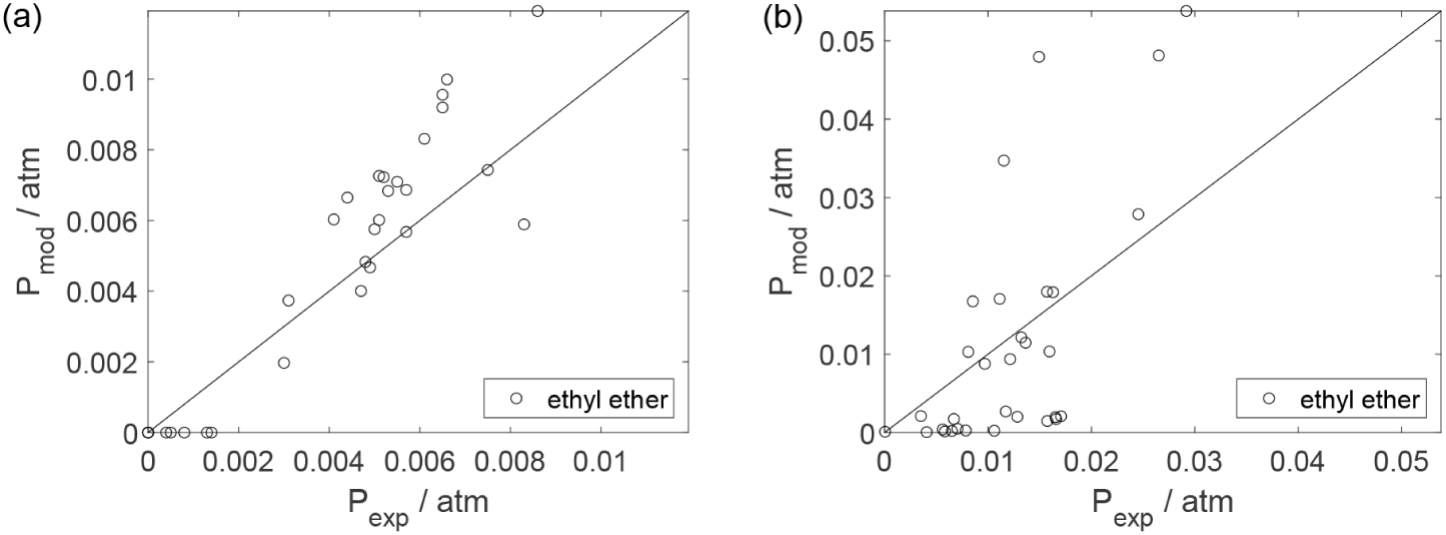
Parity plot for the partial pressure of ethyl ether: (a) experiments in the absence of oxygen; (b) experiments in the presence of oxygen; ○, experimental data; lines represent *y* = *x*.

**Figure 35:**
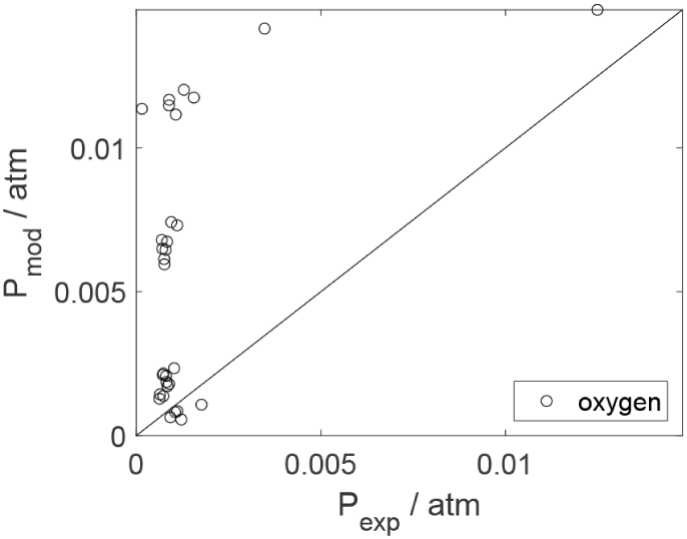
Parity plot for the partial pressure of oxygen as a function of the temperature in the experiments in the presence of oxygen; ○, experimental data; lines represent *y* = *x*.

These findings are consistent with a redox-mediated network in which low O_2_/EtOH conditions disfavor selective acetaldehyde formation because parallel dehydration and condensation pathways remain competitive at high ethanol partial pressures.

Due to practical constraints, the O_2_/EtOH window and temperatures explored here are limited; broader screening is needed to generalize the conclusions. Additionally, the present model is intentionally compact; future work should test robustness under transients and alternative feed compositions.

Recent work has shown that beyond pure-ethanol chemistry, iron–molybdate also enables the formation of acrolein from methanol/ethanol mixtures via a tandem sequence: oxidation of both alcohols to formaldehyde and acetaldehyde, followed by their aldol condensation to acrolein.^35^ Lanthanum or cerium doping tunes the acid–base properties of Fe–Mo–O and markedly promotes the condensation step, leading to acrolein yields up to ∼42% at 320 °C under MeOH/EtOH = 1 at GHSV ≈ 3900 h^–1^.^35^ This literature corroborates the bifunctional (redox/acid) nature of Fe–Mo–O that we observe under O_2_-lean operation.

## 4 Conclusions

The iron and molybdenum oxide catalyst is not selective for transforming ethanol to acetaldehyde, due to low molar oxygen-to-ethanol ratios and high partial pressures of ethanol. Accordingly, Fe–Mo–O is unsuitable for low-oxygen operation, such as above the upper flammability limit, for instance in distributed-feed reactors. This is due to the presence of parallel and series-parallel reactions that lead to the formation of ethylene, ethyl ether, ethyl acetate, and acetaldehyde derivatives, which occur at acidic and mixed sites, most likely due to the high partial pressure of ethanol. An appreciable amount of ethane formation—sensitive to the oxygen content—suggests that ethylene participates in catalyst reoxidation by removing surface hydrogen.

The structure of the catalyst varied during the tests, with an increase in specific surface area, reduction of Mo=O and Mo–O–x (*x* = Mo or Fe), and formation of β-FeMoO_4_. During the reaction, the pellets increased in size and became embrittled, indicating that the chemical-level changes affected their mechanical structure.

A reaction network was proposed to explain the reactions of dehydrogenation, dehydration, and hydrogenation in the absence of oxygen, based on the assumption that an ethoxide intermediate forms, which is common to several reactions. The data fit the kinetic model derived from this reaction network.

Future work should probe redox dynamics and quantify the role of ethylene hydrogenation to ethane under oxygen-lean operation.

## Supplementary Material



## Notes

Details of the reaction chamber of the Berty Internal Recycle Reactor, showing the turbine (Figure S1) and the internal components that are mounted inside (Figure S2) (PDF)
