# Supplementary material for: Ethanol Reactions over FeMoO Using Low O2/Ethanol Molar Ratio: Reaction Network and Kinetics
Source: ACS Omega. 2026 Jan 20;11(4):6613–27. doi: 10.1021/acsomega.5c11700 (PMC12878720; doi:10.1021/acsomega.5c11700)
Supplement: Supplementary file 1 [file ao5c11700_si_001.pdf]

Supporting Information

Ethanol Reactions over FeMoO using Low O_2/Ethanol Molar Ratio: Reaction Network and Kinetics

João G. R. Poço[1,2,*], Gustavo V. Olivieri[2], Elisabete M. Assaf[3], Reinaldo Giudici[4], Cláudio A. O. Nascimento[4]

[1]Instituto de Pesquisas Tecnológicas do Estado de São Paulo, Departamento de Nanobiomanufatura, 05508-901, São Paulo – SP – Brazil.

[2]Centro Universitário FEI, Departamento de Engenharia Química, 09850-901, São Bernardo Campo – SP – Brazil

[3]Universidade de São Paulo, Instituto de Química de São Carlos, 13566-590, São Carlos – SP – Brazil

[4]Universidade de São Paulo, Escola Politécnica, 05508-010, São Paulo – SP – Brazil

[*]Email: jgrpoco@fei.edu.br

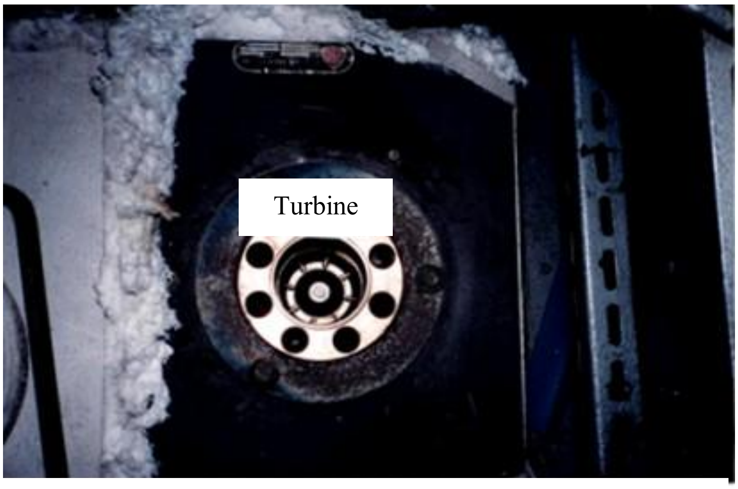

Figure S1. Internal detail of the Berty reactor.

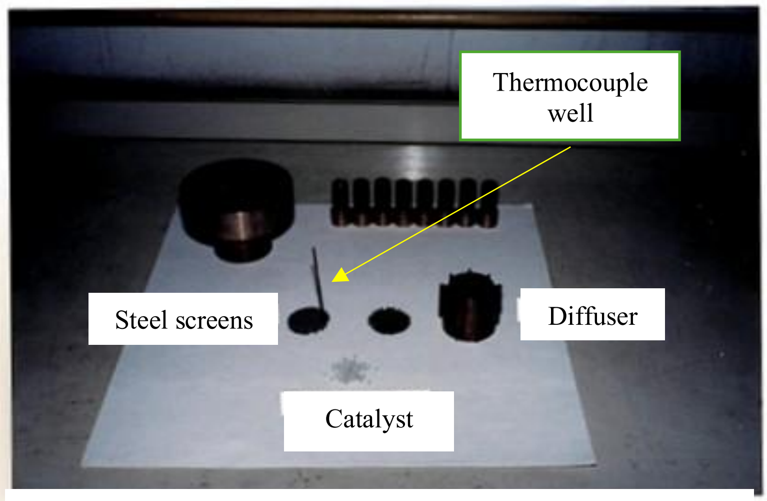

Figure S2. Internal components of the Berty reactor.
